# Cytology of malignant pleural mesothelioma: Diagnostic criteria, WHO classification updates, and immunohistochemical staining markers diagnostic value

**DOI:** 10.1002/dc.25053

**Published:** 2022-09-07

**Authors:** Nada Shaker, Douglas Wu, Abdul Majeed Abid

**Affiliations:** ^1^ Department of Pathology The Ohio State University Wexner Medical Center Columbus Ohio USA; ^2^ Department of Pathology UPMC Pittsburgh Pennsylvania USA

**Keywords:** classification, cytopathology, epithelioid, lung, mesothelioma, pleural effusion, reactive mesothelial proliferation, sarcomatoid

## Abstract

Malignant pleural mesothelioma (MPM) is a rare but aggressive malignancy with a poor prognosis. Because of this tumor rarity and overlapping histologic features with other malignancy types, the histopathological findings and diagnostic immunohistochemical workup are essential in establishing the final diagnosis of MPMs. We aimed to review the diagnostic criteria, WHO tumor classification updates, and immunohistochemical staining markers diagnostic value to achieve an appropriate clinical diagnosis.

## 1 PRIMARY OBJECTIVES

*Objective*: Morphological Features of Mesothelioma: Describe the tips for detecting malignant cells in effusion, describe the cytomorphology of mesothelioma, and indicate how these can be used to diagnose, and differentiate from reactive mesothelial proliferation and other entities, classify, sub‐type and discuss the risk factors for diffuse malignant mesothelioma.

## 2 SECONDARY OBJECTIVES

*Objective 1*: Gross description: Discuss tissue specimen obtaining, volume, and processing.

*Objective 2*: Grading and staging: Discuss how the grading system for epithelioid malignant pleural mesothelioma (MPM) provides prognostic stratification.

*Objective 3*: Radiologic finding: Discuss the available imaging modalities for the evaluation of malignant pleural mesothelioma.

*Competency*: Diagnostic medicine and therapeutic: Discuss the use of diagnostic and predictive IHC and molecular assays.

## 3 CLINICAL PRESENTATION (EDUCATIONAL CASE)

A 63‐year‐old male former carpenter was referred to the respiratory clinic by his primary care physician with discomfort in her chest and upper abdomen for the last 3 months. Symptoms associated with dyspnea, dry bloody cough, fatigue, and weight loss of 20 lbs. Physical examination reveals dullness on percussion and decreased breath sounds on auscultation of the right lung, hemoptysis, ascites, and pedal edema. The suspicious radiological finding of right‐sided pleural effusion and thickening of the pleura around the effusion has been detected on chest X‐ray. The patient worked in a shipyard for 30 years and was involved in the construction, repair, and demolition of vessels. He often worked below deck in enclosed environments, where he was exposed to visible clouds of asbestos dust.

The patient had no significant past medical history. He had never smoked cigarettes, although he had occasionally smoked cigars and pipes in the past and described a history of social alcohol use. There is no family history of cancer.

## 4 DIAGNOSTIC FINDINGS

Studies of the patient ventilatory function included forced vital capacity (FVC), forced expiratory volume in 1 s (FEV1), total lung capacity (TLC), residual volume (RV), calculated FEV1/FVC, and a single breath diffusing capacity for carbon monoxide (DLCO). These tests showed a moderate restrictive defect with a normal diffusing capacity.

Chest X‐ray revealed a right‐sided pleural effusion and circumferential nodular pleural thickening and thickening of the interlobular septa.

The chest X‐ray is usually followed by a contrast‐enhanced chest CT scan. A chest computed tomography (CT) showed intralobular, small, rounded, and branching opacities; thickened interlobular septa; subpleural curvilinear lines; and parenchymal bands. Honeycombing is seen. Suspicious for malignancy is reported.

## 5 IMPORTANT CLUES

### 5.1 What is the etiology, clinical presentation, differential diagnosis, and clinical approach for the diagnosis of pleural effusion?

Pleural effusion is a common diagnosis in clinical medical practice and the differential diagnosis is wide. Precise differential diagnostic categorization is essential for guiding the treatment and assessing the prognosis of the pleural effusion.^1^

The most common causes of pleural effusion are congestive heart failure, malignancy, pneumonia, and pulmonary embolism.^1^ Patient with pleural effusion can be asymptomatic or can present with progressive dyspnea, cough, hemoptysis, upper abdominal pain, and pleuritic chest pain.^1^

The foundation of the further diagnostic work‐up is the thoracocentesis (pleural tap) which enables the differentiation of a transudate from an exudate.^2^ A standard panel of tests includes Pleural Fluid protein, glucose, pH, lactate dehydrogenase (LDH), cytology, and microbiology.^2^ Table 1 shows the differential diagnosis of pleural fluids.

**TABLE 1:** Differential diagnosis of pleural fluids

Pleural fluids results	Common causes
Low glucose and/or high LDH	Pleural infection, rheumatoid effusion, esophageal rupture, lupus pleuritis
High protein levels	Exudative effusion, high levels are often caused by tuberculosis
Exceptionally low protein (<15 g/dl)	Dural leak
Lymphocyte predominance	TPE, lymphoma, post cardiac bypass graft, renal or liver failure, rheumatoid arthritis
Neutrophil predominance	parapneumonic effusions, early tuberculous pleural effusion, less commonly due to pulmonary embolism or pancreatitis
Eosinophil predominance	Malignant and parapneumonic effusions

### 5.2 Transudate versus exudate

The common causes of transudative pleural effusion include cirrhosis, hypoalbuminemia, and peritoneal dialysis. Rarer causes include hypothyroidism, nephrotic syndrome, constrictive pericarditis, urinothorax, and Meigs' syndrome.^2^ An exudative effusion, conversely, is usually caused by a localized disease to the pleura such as infection (including tuberculosis), autoimmune disease, pulmonary embolism, and malignancy.^2^

### 5.3 Light's criteria

In 1972, Dr. Richard Light published a study producing criteria that have a high sensitivity of 99.5% and a high specificity for differentiating transudative from exudative effusions in 93% to 96% of cases.^3^ Light criteria to diagnose a biochemically exudative effusion require one or more of the following: Pleural fluid protein to serum protein more than 0.5, pleural fluid LDH to serum LDH more than 0.6, or pleural LDH more than two‐thirds of the upper limit of the normal serum LDH level. Any one of these criteria being present predicts an exudative effusion with an accuracy reaching 94.7%.^3^

### 5.4 What should be done if the initial evaluation is nondiagnostic or suspected of a malignancy?

The initial assessment of the patient with pleural effusion should include an ultrasonography‐guided thoracentesis to obtain specimens to categorize the effusion as a transudate or an exudate and to examine for microbiology and cytology.^4^ Since the initial assessment with thoracentesis and a computed tomographic scan cannot rule out malignant cases or tuberculosis disease, a pleural biopsy is required to identify recurrent and unidentified causes of exudative effusions.^4^

Pleural biopsy (e.g., video‐assisted thoracoscopic surgery [preferred], CT‐guided core biopsy, open biopsy, and mediastinoscopy) with lymph node sampling are the gold standard for the diagnosis of malignant pleural effusions and assessing the extent of the tumor.^4^

MRI is used to better delineate the relationship of malignant pleural mesothelioma to adjacent structures and organs. PET/CT might help to identify metastatic disease.^5^

Serology for mesothelin and fibulin‐3 might be useful as screening markers for malignant pleural mesothelioma, osteopenia lacks specificity.^6^

### 5.5 What is the radiologic finding in the malignant pleural mesothelioma?

Chest X‐ray can reveal unilateral effusion. CT scan with contrast enhancement helps in detecting the unilateral pleural effusion, the loculated, nodular, or diffuse pleural thickening, assessing the involvement of visceral, parietal, and diaphragmatic pleura, and identifying the possible extension into fissures.^5^

#### 5.5.1 A classic example of the radiologic finding is an educational case

A 63‐year‐old male presented with progressive exertional dyspnea and dry cough. Frontal chest radiography shows a right‐sided pleural effusion. CT scan shows a Right‐sided moderate pleural effusion. CT scan shows an irregular thickening of pleura throughout the right hemithorax.

### 5.6 What lesions can arise in the pleura?

Benign lesions of the pleura are relatively common and usually detected incidentally. These include pleural lipomas, pleural plaques, and diffuse pleural thickening.^7^

Malignant tumors of the pleura include 10%–20% cases of solitary fibrous tumors of the pleura and malignant pleural mesothelioma.^7^

Pleural lipomas are benign, slow‐growing tumors that arise from fat cells of the pleural lining. They are well circumscribed and can typically be differentiated from their malignant counterpart, liposarcoma, which tends to have a heterogeneous appearance on imaging.

Pleural plaques and pleural thickening, although not typically a tumor, represent a reaction to environmental irritants and can mimic malignancy. With prolonged asbestos exposure, bilateral pleural plaques can sometimes visibly calcify. Diffuse pleural thickening is a more extensive disease process involving the lung parenchyma.^7^

Solitary fibrous tumors of the pleura are discovered as an incidental radiographic finding. They can grow exceptionally large and cause severe symptoms such as chest pain, cough, and dyspnea from the mass effect. While solitary fibrous tumors are most likely benign, they can be malignant in 10%–20% of cases.^8^ Malignant pleural mesotheliomas are rare tumors arising from mesothelial cells of the pleura and peritoneum.

### 5.7 What is the epidemiology of malignant pleural mesothelioma?

Malignant pleural mesothelioma (MPM) is a rare malignancy with a poor prognosis, linked to asbestos exposure in 54%–90% of patients. The pericardium and tunica vaginalis testis are Infrequent sites of origin. More than 80% of mesotheliomas are localized in the pleura. Men are more frequently affected than women. The median age is >60 years.^9^

Pleural implants caused by metastatic disease from another origin with the main primaries from the lung, breast, and ovarian cancer, and thymoma and mesothelioma locally should be differentiated from malignant pleural mesothelioma.^10^ The use of asbestos became widespread in recent years with increased industrialization as it is used as a form of insulation in many industries. These include construction, shipbuilding, pipefitting, and car brake assembly. Asbestos is a natural silicate mineral categorized as serpentine and amphibole. The amphibole shape of asbestos is believed to cause chronic irritation that may lead to malignant transformation of the pleura with the repeated exposure.^11^

### 5.8 Describe the cytomorphologic features of mesothelioma and how would you differentiate it from reactive mesothelial proliferation and other entities?

**FIGURE 1:**
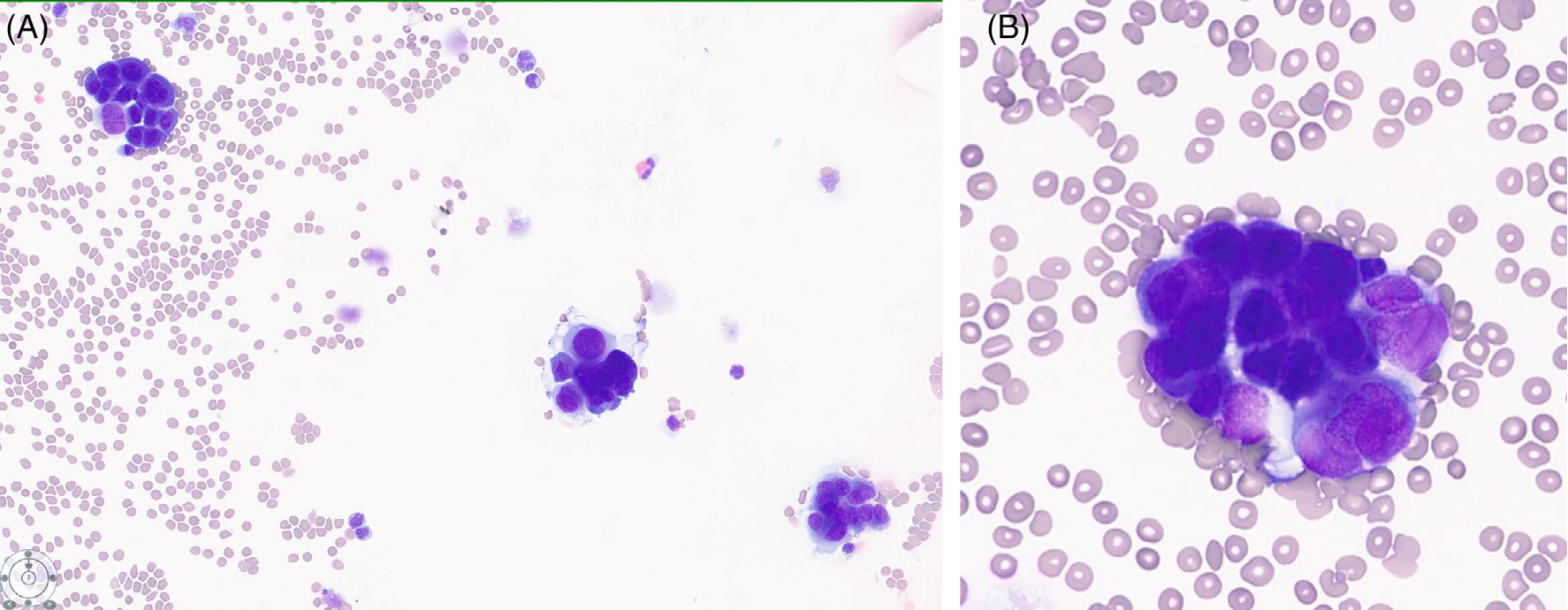
(A) Peritoneal mesothelioma Diff‐Quik stained aspirate smear (20×). (B) Peritoneal mesothelioma Diff‐Quik stained aspirate smear 40×. Note the presence of large clusters of epithelioid cells with knobby borders, and morulae [Colour figure can be viewed at wileyonlinelibrary.com]

The histomorphology criteria of malignant pleural mesothelioma have been well documented recently in the WHO “Blue Book.”^12^ The pathohistological differentiation of malignant pleural mesothelioma from atypical reactive mesothelial proliferation, adenocarcinoma, squamous cell carcinoma, epithelial hemangioendothelioma, and epithelioid angiosarcoma can be a diagnostic challenge.^12^ Key features for detecting malignant cells in effusion include “second population,” numerous large clusters, necrosis, and lacunae. A good way to identify malignant cells in effusions is first to locate some benign mesothelial cells “the second population.” Although some malignant cells are not necessarily larger than mesothelial cells, malignant cells can be differentiated from adenocarcinoma by the presence of cell groups with knobby borders (“morulae”), low nuclear: cytoplasmic ratios, lack of pleomorphism, and dense cytoplasm.^13^ Key cytologic criteria features that are essential for the diagnosis of malignant mesothelioma include reactive mesothelial hyperplasia with macro nucleoli and large cell clusters with more than 12 cells.^13^ Figure 1A,B.

### 5.9 What is the updated 2015 WHO classification of malignant pleural mesothelioma? What is the major change in the forthcoming WHO classification?

2015 WHO classification of pleural mesotheliomas includes three major histologic subtypes‐epithelioid, sarcomatoid, and biphasic.^14^ Because of tumor rarity and overlapping histologic features, recent genomic data has supported the need for a more granular and clinically valid classification beyond the three current subtypes.^14^ The major change in the forthcoming WHO classification is the inclusion of mesothelioma in situ as a diagnostic category.^14^ Table 2 shows the updated 2015 WHO Classification of Mesothelioma.

**TABLE 2:** 2015 WHO classification of mesothelioma

Tumor type	Characterizations
Epithelioid mesothelioma	Composed of rounded rather than spindle‐shaped cells usually showing a cohesive architecture, although epithelioid cells can show single cell growth within fibrous stroma.
Sarcomatoid mesothelioma, including desmoplastic variant	Composed of spindle‐shaped (greater than two times longer than wide). The spindle cells may lie in varying amounts of fibrous stroma, or they can form solid sheets.
Biphasic mesothelioma	Showing at least 10% of both epithelioid and sarcomatoid morphology. This rule is limited to definitive resections, namely, extended pleurectomy/decortication and extra pleural pneumonectomy. For smaller samples, until more data are collected, the group proposes that the diagnosis of “biphasic” can be rendered regardless of the percentages of each component present and that the diagnosis should be accompanied by a comment indicating the percentages of each component.
Well‐differentiated papillary mesothelioma	A rare localized mesothelial neoplasm characterized by an exophytic papillary architecture lined by relatively bland mesothelium with no or only minimal areas of invasion. Diagnosis requires exclusion of diffuse malignant mesothelioma with papillary architecture.
Adenomatoid tumor	Rare and benign mesothelial tumors arise from the lining of organs. It mainly presents in the genital tract
Diffuse and localized mesothelioma	Vast majority of diagnosed cases are diffuse mesotheliomas, meaning that the tumors are small, multiple, and spread out over a significant area and multiple parts of the body. Localized tumors are fewer in number with well‐defined boundaries. They tend to be localized to limited area of an organ

### 5.10 What is the role of diagnostic and predictive immunohistochemical stains and molecular assays testing in the diagnosis of malignant pleural mesothelioma?

**FIGURE 2:**
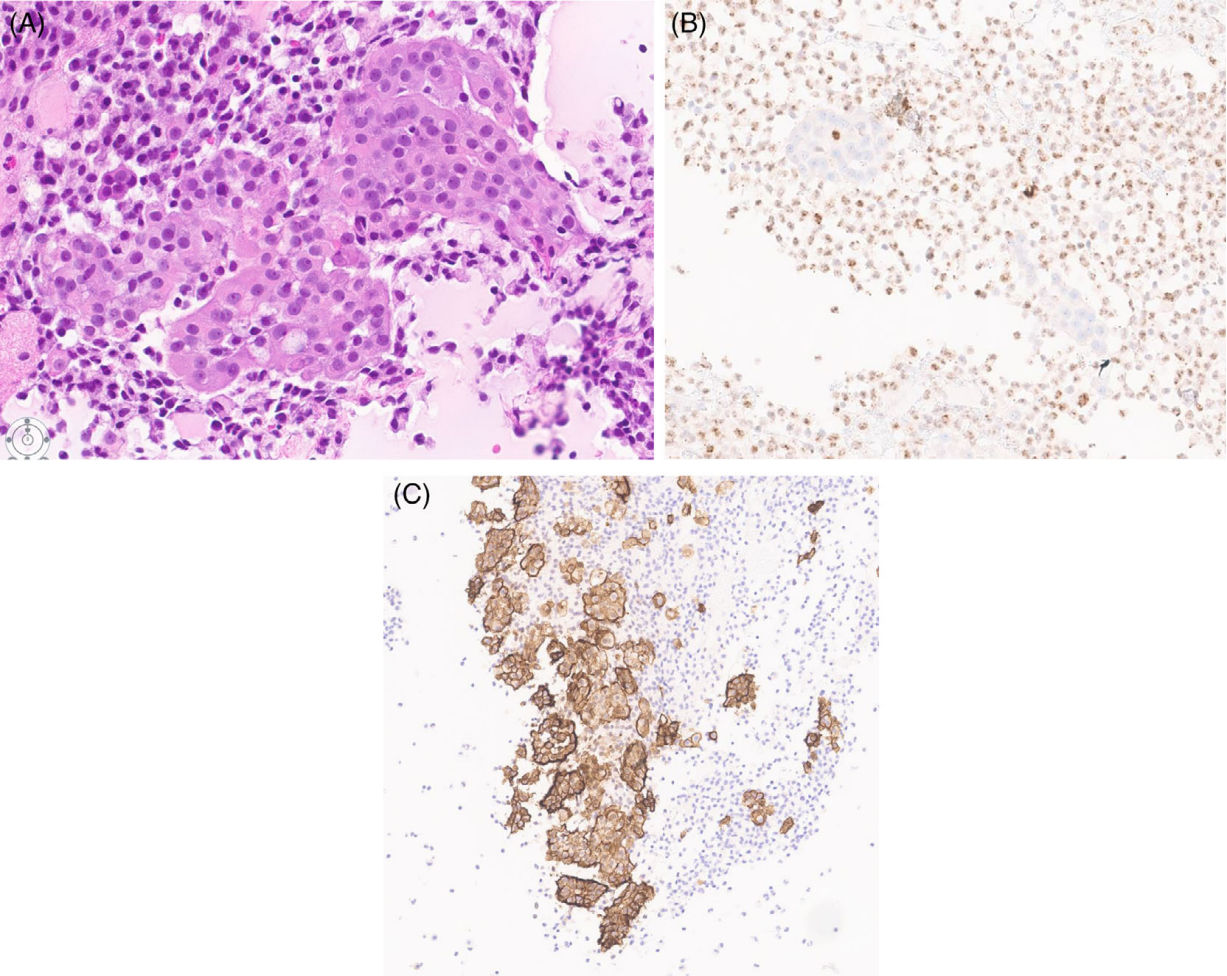
(A) Hematoxylin & Eosin‐stained cell block section (20×). (B) Loss of BAP1 on cell block section (17.5×). (C) D2‐40 marker on cell block section (11×) [Colour figure can be viewed at wileyonlinelibrary.com]

The utility of Immunohistochemical stains analysis to confirm the mesothelial origin and exclude adenocarcinoma from different origins is well studied.^15^ Wilms tumor 1 (WT1)/AE1/AE3, claudin 4, and BRCA1‐associated protein 1 (BAP1) immunostains are useful new tools that have been proposed for the distinction between Malignant Mesothelioma and benign mesothelial reactions.^15^ Loss of expression of BRCA1 associated protein (BAP1) or methylthioadenosine phosphorylase (MTAP) or homozygous deletion of cyclin‐dependent kinase inhibitor 2A (CDKN2A) (p16) by FISH helps to distinguish reactive mesothelial cells from malignant pleural mesothelioma. IHC staining for BAP1 is lost in a substantial subset of malignant mesotheliomas but retained in benign mesothelial proliferations.^16^ The use of BAP1 and CDKN2A/MTAP improves the diagnostic sensitivity of effusion specimens and is valuable in establishing the diagnosis of epithelioid mesothelioma.^16^ Figure 2A,B,C.

An important differential diagnosis to take into consideration when differentiating malignant pleural mesothelioma is serous carcinoma of the gynecologic tract, both of which are WT1 positive. BAP1 is retained in almost all serous carcinomas,^16^ making it useful to differentiate between malignant mesothelioma and serous carcinoma, additional panel could include paired box 8 (PAX8) (usually positive in serous carcinoma) and calretinin (usually positive in mesothelial cells). Recent studies suggest the diagnostic utility of D2‐40, a novel lymphatic marker for malignant mesothelioma. The diagnostic utility of D2‐40 marker is yet to be evaluated. In addition, this marker interpretation should be done with caution due to its occasional weak positivity. This might also be challenging in limited cellularity specimens. A combined immunohistochemical staining panel with other markers seems to be essential to acquire accurate diagnostic results.^17^, ^18^

## FUNDING INFORMATION

This project was funded by the Ohio State University.

## CONFLICT OF INTEREST

The authors declare no potential conflict of interest.

## Data Availability

The data that support the findings of this study are available on request from the corresponding author. The data are not publicly available due to privacy or ethical restrictions.
